# Effects of Selected Functional Bacteria on Maize Growth and Nutrient Use Efficiency

**DOI:** 10.3390/microorganisms8060854

**Published:** 2020-06-05

**Authors:** Amelia Tang, Ahmed Osumanu Haruna, Nik Muhamad Ab. Majid, Mohamadu Boyie Jalloh

**Affiliations:** 1 Faculty of Agriculture and Food Sciences, Universiti Putra Malaysia Bintulu Sarawak Campus, Bintulu 97008, Sarawak, Malaysia; ameliatang18@gmail.com; 2 Institute of Tropical Agriculture and Food Security (ITAFoS), Universiti Putra Malaysia, Serdang 43400, Selangor, Malaysia; 3 Institute of Tropical Forestry and Forest Products (INTROP), Universiti Putra Malaysia, Serdang 43400, Selangor, Malaysia; nik@upm.edu.my; 4 Faculty of Sustainable Agriculture, Universiti Malaysia Sabah, Sandakan Branch, Locked Bag No. 3, Sandakan 90509, Sabah, Malaysia; mbjalloh@ums.edu.my

**Keywords:** *Paraburkholderia nodosa*, *Burkholderia cepacia*, *Serratia nematodiphila*, PGPRs, integrated nutrient management

## Abstract

Plant growth-promoting rhizobacteria (PGPR), which include isolates from genera *Paraburkholderia*, *Burkholderia* and *Serratia,* have received attention due to their numerous plant growth-promoting mechanisms such as their ability to solubilize insoluble phosphates and nitrogen-fixation. However, there is a dearth of information on the potential plant growth-promoting effects of these three groups of bacteria on non-legumes such as maize. This study determined the influences of the aforementioned strains on soil properties, maize growth, nutrient uptake and nutrient use efficiency. A pot trial using maize as a test crop was done using a randomized complete block design with 7 treatments each replicated 7 times. The treatments used in this study were: Control (no fertilizer), chemical fertilizer (CF), organic-chemical fertilizers combination without inoculum (OCF) and with inocula consisting of single strains [cellulolytic bacteria (TC), organic fertilizer and chemical fertilizer with N-fixing bacteria (TN), organic fertilizer and chemical fertilizer with P-solubilizing bacteria (TP)) and three-strain inocula (TCNP), respectively. The variables measured included plant growth and nutrient content, soil nutrient content and functional rhizospheric bacterial populations. *Paraburkholderia nodosa* NB1 and *Burkholderia cepacia* PB3 showed comparable effects on maize biomass and also improved N and P use efficiencies when compared to full chemical fertilization. Nitrogen-fixing rhizobacteria had a positive effect on above-ground biomass of maize. *Paraburkholderia nodosa* NB1 improved soil total C and organic matter contents, besides being the only bacterial treatment that improved K use efficiency compared to OCF. The results suggest that *P. nodosa* NB1 and *B. cepacia* PB3 have potential usage in bio-fertilizers. In contrast, treatments with *Serratia nematodiphila* C46d and consortium strains showed poorer maize nutrient uptake and use efficiency than the other single strain treatments. Bacterial treatments generally showed comparable or higher overall N and P use efficiencies than full chemical fertilization. These findings suggest that at least half the amounts of N and P fertilizers could be reduced through the use of combined fertilization together with beneficial bacteria.

## 1. Introduction

Soil fertility and nutrient cycling are influenced by the soil microbial population. An understanding of the interrelationships between functional microbial communities and cycling of C, N, P and S as an indicator of soil health is essential for soil fertility management [1]. The major shortcomings associated with the use of chemical fertilizers include high cost, limited supply and environmental polluting effects (especially from N and P fertilizers). Thus, there is a need for alternative and sustainable sources of nutrients for crops. The adoption of an integrated nutrient management (INM) approach offers an alternative strategy against the adverse effects of using conventional N and P fertilizers alone. An INM system involves minimal chemical fertilizer application in combination with both natural and man-made sources of crop nutrients to improve nutrient-use efficiency and crop productivity in an environmental-friendly manner [2]. In addition, advances in the understanding of the relationships between crops and microorganisms colonizing rhizospheric soils have increased screening efforts for microbial strains showing plant growth-promoting (PGP) ability. This ability is through direct and/or indirect enhancement of plant nutrient uptake by use as microbial inoculants or in microbial-based fertilizers termed as bio-fertilizers.

Plant growth-promoting rhizobacteria (PGPR) are free-living soil bacteria which when applied to seed or crops, propagate aggressively in the rhizosphere or plant roots and impact crop growth and production [3]. Thus, beneficial crop-microbe interactions in the rhizosphere could improve crop vigor and soil fertility [4]. Most PGP effects of PGPR are by means of metabolite production that perfunctorily enhance growth. Thus, PGPR influence crop growth and development by modifying the physiological conditions and morphological characteristics of inoculated roots to improve nutrient uptake [5,6,7,8]. As an example, plants treated with *Azospirillum* exhibited increased N, P, K use efficiency and microelements uptake [9]. Bio-fertilizers exert better crop growth effects via diverse or a combination of mechanisms which include improved soil nutrient availability, biological N_2_ fixation, phytohormone production and disease antagonism [10].

The genus *Burkholderia* comprises diverse species and many strains have been found in human and animal samples in addition to environmental sources [11]. The plant growth-promoting rhizobacteria status of many *Burkholderia* species has been well established because of the many PGP mechanisms shown by the strains. Some have been reported to possess N-fixing ability as free-living or endophytic bacteria [12] or by forming a symbiotic relationship with leguminous plants [13,14]. Some strains have been reported to be phosphate-solubilizing bacteria (PSB) [15,16]. Others with the ability to inhibit many plant-disease-causing fungi are being used as biocontrol agents [16,17]. Among other known features of the strains of this genus include phytohormone and/or siderophore production [18]. However, the genus *Burkholderia* has recently been divided into two genera with the novel genus *Paraburkholderia* [19,20,21] bearing primarily environmental *Burkholderia* species which are cited as the good or beneficial ones [19,22,23]. *Burkholderia sensu stricto* genus consists of environmental, clinical and phytopathogenic species that are harmful [19,24]. 

*Paraburkholderia nodosa* strains reported as legume-symbionts have been isolated from root nodules of *Mimosa* (*M. bimucronata* and *M. scabrella*) [25] and other plants from the “Piptadenia group” [26]. More recently *P. nodosa* strains were also recovered from members of the *Papilionoidea* subfamily such as in *Phaseolus vulgaris* [27]. However, information on free-living *P. nodosa* strains and their potential PGP effects on non-legume plants such as maize is lacking.

The environmental *Burkholderia cepacia* is classified as one of the *Burkholderia cepacia* complex (Bcc) strains, in which the latter are opportunistic pathogens in patients with cystic fibrosis and immuno-compromised individuals [28]. The Bcc strains have since been severely restricted in agricultural usage [28]. Nevertheless, many *Burkholderia cepacia* strains have been reported to possess considerable biotechnological potentials such as PGPR [29] and biological control of plant pathogens [30,31]. In terms of phosphate-solubilization, many studies have found *Burkholderia cepacia* strains to be responsible for P release from polyphosphate in low pH environments [32,33,34,35]. Similarly, *Burkholderia cepacia*, a PGPR strain isolated from Korean agricultural soils solubilized insoluble phosphates, owing to its large production of organic acids especially gluconic acid [36].

The genus *Serratia* belongs to the family *Enterobacteriaceae* of the class *Gammaproteobacteria*. Although some members of this genus, in particularly *S. marcescens,* have clinical importance as human pathogens [37,38], many *Serratia* species were found to be plant-associated beneficial bacteria [39]. For instance, *S. plymuthica* enhanced the growth of plants and inhibited soil-borne plant pathogens [40]; *S. liquefaciens*, *S. plymuthica* and *S. rubidaea* associated with the rhizosphere of oilseed rape was shown to have antifungal properties [41] and an association of *S. rubidaea* with marine alga [42]. Furthermore, *S. nematodiphila*, along with *S. entomophila*, *S. glossinae*, *S. proteamaculans* and *S. ureilytica* have been reported as non-pathogenic to humans [39].

The first report on novel *Serratia nematodiphila* was of the strain symbiont with the entomopathogenic nematode *Heterorhabditidoides chongmingensis* (Rhabditida: Rhabditidae) and it was isolated from the latter’s intestine [43]. The *Serratia nematodiphila* strain was non-cellulolytic [44]. There have been many reports on the beneficial effects of *Serratia nematodiphila* such as a multiple-PGPR-trait strain improving black pepper plant growth [4], gibberellin-producing-PGPR strain improving growth of pepper plants under low-temperature stress [45] and an endophytic bacterial strain LRE07 reducing growth suppression of *Solanum nigrum* L. grown in a cadmium-polluted soil [46]. However, there is a dearth of information on the cellulolytic strain *Serratia nematodiphila* as PGPR and its effects on maize plants.

There are several research reports on the beneficial impacts of PGPR on maize growth. Bacterial genera such as *Azospirillum*, *Azotobacter*, *Acetobacter*, *Pseudomonas*, *Paraburkholderia*, *Herbaspirillum* and *Rhizobium* have been reported to show good PGPR effects on maize. Noumavo et al. [47] found that *Azospirillum lipoferum*-treated plants produced maximal plant heights and underground biomass by 37.32% and 56%, respectively, whereas a combination of *Pseudomonas fluorescens* and *P*. *putida* produced higher aerial dry matter by 59.11% compared with the control. Co-inoculation of *Bacillus megaterium*, *Azotobacter chroococcum* and *Bacillus mucilaginous* has also been reported to significantly improve maize biomass and height with comparable effects 50% higher than that with chemical fertilizer alone [48].

Therefore, the objectives of this study were to determine the effects of beneficial cellulolytic *Serratia nematodiphila* C46d, N-fixing *Paraburkholderia nodosa* NB1 and phosphate-solubilizing *Burkholderia cepacia* PB3 on: (1) interactions among functional rhizobacteria, (2) selected growth variables of maize, (3) plant growth-promotion based on nutrient uptake and use efficiency of maize and (4) selected properties of the Bekenu series soil. It was hypothesized that the right amount of chemical fertilizer and compost combination with selected PGPR inoculants in single or consortium treatment used in this present study could exert positive influences on maize growth and nutrient use efficiency on Bekenu series (*Typic Paleudults*).

## 2. Materials and Methods

### 2.1. Bacterial Inoculants

Bacterial cultures of *Serratia nematodiphila* C46d, *Paraburkholderia nodosa* NB1 and *Burkholderia cepacia* PB3 isolated from a rehabilitated forest soils at Universiti Putra Malaysia Bintulu Sarawak Campus, Malaysia were used in this study. All the isolates were previously identified based on 16S rRNA gene sequence similarities and were also characterized based on morphological and biochemical properties [49] using Bergey’s Manual of Determinative Bacteriology and Bergey’s Manual of Systematic Bacteriology [50,51]. 

The functional and cross-functional activity indices of the three isolates were calculated by ratio of total diameter (colony + halo or clearing zone around colonies) and colony diameter [52] on selective media.

Phosphate-solubilization activity was evaluated on tricalcium phosphate medium (modified from Nautiyal [53], Buis [54], Gupta et al. [55] and Kokal [56]): Prepared using a 50 mL salts solution (containing 100.0 g L^−1^ MgCl_2_·6H_2_O, 5.0 g L^−1^ MgSO_4_·7H_2_O, 4.0 g L^−1^ KCl and 2.0 g L^−1^ (NH_4_)_2_SO_4_), 10.0 g sucrose, 2.5 g Ca_3_(PO_4_)_2_, 6 mL bromophenol blue (in 0.4% ethanol solution), 15.0 g agar, 1.0 L distilled water, pH 7.0. Cellulolytic activity was evaluated on cellulose-Congo red medium (modified from Hendricks et al. [57]): Prepared using 0.50 g K_2_HPO_4_, 0.25 g MgSO_4_, 1.88 g cellulose microgranular powder, 0.20 g Congo red (after pH modification), 15.0 g agar, 2.0 g gelatine, 100 mL soil extract (1:2 soil solution ratio), 900 mL tap water, pH 7.0. Nitrogen-fixation activity was evaluated on N-free malate (Nfb) medium (modified from Döbereiner and Day [58]): Prepared using 0.4 g KH_2_PO_4_, 0.1 g K_2_HPO_4_, 0.2 g MgSO_4_.7H_2_O, 0.1 g NaCl, 0.02 g CaCl_2_, 0.01 g FeCl_3_, 0.002 g Mo.O_4_Na.2H_2_O, 5.0 g sodium malate, 5 mL bromothymol blue (in 0.5% alcohol solution), 15.0 g agar, 1.0 L distilled water, pH 7.0. 

The three isolates possess phosphate-solubilizing ability with the phosphate-solubilizing isolate *Burkholderia cepacia* PB3 exhibiting a phosphate-solubilization activity index value of 4.93, cellulolytic activity of 4.58 and N-fixing index value of 3.12. Cellulolytic isolate *Serratia nematodiphila* C46d demonstrated a functional activity of 10.41 on cellulose agar media, phosphate-solubilization activity of 2.10 and 0.00 (zero) N-fixing ability. Nitrogen-fixing isolate *Paraburkholderia nodosa* NB1 without cellulose-hydrolyzing capability had the lowest phosphate-solubilization activity index of 1.41 and showed the strongest intensity blue halo on Nfb medium with a N-fixing index value of 10.82 among all three isolates. These three isolates also produced phytohormone indole-3-acetic acid (IAA) which was determined according to Asghar et al. [59], with *Serratia nematodiphila* C46d, *Burkholderia cepacia* PB3 and *Paraburkholderia nodosa* NB1 generating IAA quantities of 11.39 µg mL^−1^, 7.81 µg mL^−1^ and 0.43 µg mL^−1^, respectively.

### 2.2. Bacterial Compatibility or Interactions and Establishment of Bacterial Exponential Growth Equations

The three selected bacterial isolates used as inocula in this pot trial were observed for their positive or negative interactions with one another via cross streak assay method using nutrient agar medium [60]. Absence of any inhibition zone at crossover areas is an indication of compatibility or non-antagonistic interaction among the tested isolates.

The growth of the aforementioned three bacterial isolates was studied so as to establish a modeling equation unique to each isolate for estimation of their population exponential growth at any given time. A few colonies from a purified plate culture were inoculated into 50 mL of nutrient broth in a 250 mL Erlenmeyer flask before agitating at 200 rpm at 30 °C. At 1 hr interval, starting from 0 hr or before beginning the assay by agitation, a portion of the well-mixed culture was dispensed for absorbance reading using a UV–Vis spectrophotometer at 600 nm and serial dilutions before plating on nutrient agar for colony-forming-unit (cfu) counting. The procedure was repeated hourly up to stationary phase as indicated by the observed trend of optical density (OD) values. Samples were diluted to be within the linear range of the dynamic optical system. The number of colonies formed on the plates was counted after 16 hrs of incubation or as soon as colonies were visible for counting. The cfu counts were plotted against OD readings (which were both selected at up to log growth phase) in triplicate datasets using a curve-fitting software (DataFit Version 9.0 by Oakdale Engineering) and each selected isolate‘s exponential growth equation was established.

### 2.3. Modulation and Preparation of Solid Carriers as Inocula

Two types of carrier formulations or materials were shortlisted to be optimized and tested, after which one was selected for the bacterial inoculation in the pot trial. The materials were peat moss and compost-charcoal powder mixture at a ratio 3:1. The peat moss was Tulip Profi No 1 from Hardenberg, Netherlands. Both formulations were oven dried at 70 °C for 72 hr, ground and sieved to pass a 2-mm sieve. The water-holding capacity of both formulations was determined so as to ascertain the maximum volume of bacterial culture to be inoculated or mixed into the carriers. Five grams of each carrier type was packed in small autoclavable plastic bags and amended with approximately 10% CaCO_3_ to maintain a neutral pH. Twenty percent gum arabic was added at 1 mL g^−1^ as carrier stabilizer and all components were then mixed well manually. They were then autoclaved at 121 °C for 20 min.

The three selected bacterial isolates NB1, PB3 and C46d were used as inoculants. Their cultures were prepared by inoculating each isolate’s pure plate culture colonies into 100 mL nutrient broth in 250 mL Erlenmeyer flasks. The flasks were then agitated at 200 rpm at 30 °C with several intermittent samplings in aseptic conditions for optical density checks, up to log phase of growth before harvesting. The bacteria were harvested by centrifugation at 6000 rpm at 4 °C before being aseptically transferred into both types of carriers to up to 50% water retention capacity level of each carrier type. The mean population sizes of NB1, PB3 and C46d were determined to be 9.78 × 10^6^ cfu mL^−1^ g^−1^, 3.64 × 10^8^ cfu mL^−1^ g^−1^ and 4.96 × 10^8^ cfu mL^−1^ g^−1^, respectively following inoculation.

The inoculant-carriers were manually mixed before aseptically resealing the inocula packets. These were then placed for curing at room temperature and away from sunlight or heat for 3 days, followed by quality check via cfu counting. After the curing period the rest of the carrier-inoculants were transferred into a refrigerator for storage at 4 °C for further use with maximum storage time of 3 months. The inoculated peat moss was chosen as the carrier-inoculant for the pot trial based on its higher cfu counts by one logarithm compared to the compost-charcoal formulation after the curing period and prior to use. One day before the commencement of inoculation for the pot trial, the peat moss inoculums were incubated overnight at room temperature in the dark to allow bacterial cells to equilibrate or adjust to the higher temperature at the pot trial site. The compost used for the carrier formulations and the pot trial was locally supplied by Green Grow, Malaysia. The total N, available P and available K contents of the compost were 0.23%, 87.1 mg kg^−1^ and 714 mg kg^−1^, respectively.

### 2.4. Soil Characterization

The soil used for the pot trial was Bekenu Series (*Typic Paleudults*). It was collected at 0 to 25 cm depth using an auger from an uncultivated area at Universiti Putra Malaysia Bintulu Sarawak Campus, Sarawak, Malaysia. The soil samples were air-dried, ground to pass a 2-mm sieve and mixed thoroughly. Soil bulk density and texture were determined using the methods according to Tan [61]. The following soil properties were analyzed before and after the pot trial. Soil pH in distilled water and 1M KCl solution (both with a soil and solution ratio of 1:2.5) were determined using a pH meter [62]; organic matter and total C via combustion method [63]; total N using Kjeldahl method [64]; and exchangeable NH_4_^+^ and available NO_3_^-^ using Keeney and Nelson [65] method. The soil available P and exchangeable cations (K, Ca and Mg) were extracted using double acid method [66]. Then the concentration of soil available P was determined using the molybdenum blue method [67] and Atomic Absorption Spectrophotometry (AAS) (Analyst 800, Perkin Elmer, Norwalk, USA) was used for the exchangeable cations. Soil CEC was determined by leaching method [68] followed by steam distillation [64]. The bulk density of the soil was 1.10 g cm^−3^ and a sandy clay loam texture. Selected chemical properties of the soil are shown in Table A1 [69].

### 2.5. Pot Trial

A pot trial was conducted in a greenhouse at Universiti Putra Malaysia Bintulu Sarawak Campus, Malaysia using randomized complete block design (RCBD). The experimental site experiences an average temperature of 31.90 ± 3.20 °C, relative humidity of 69.40 ± 13.70% and light intensity of 34,500.00 ± 6362.10 lux. The plastic pots used in this study were sized Ø_bottom_ 19 cm × 23 cm x Ø_top_ 28 cm and each was filled with 7.15 kg soil (adjusted based on the bulk density of the soil and the size of the pots). Seven treatments including bacterial inoculations and uninoculated treatments were used. The treatments were: organic fertilizer and chemical fertilizer without any bacteria (OCF), organic fertilizer and chemical fertilizer with cellulolytic bacteria (TC), organic fertilizer and chemical fertilizer with N-fixing bacteria (TN), organic fertilizer and chemical fertilizer with P-solubilizing bacteria (TP), organic fertilizer and chemical fertilizer with the three bacterial types (TCNP), chemical fertilizer alone (CF) and no treatment at all (Control). The details and the codes of the treatments are shown in Table 1.

Maize (var. F1 Thai hybrid) was used as the test crop. Fertilizer requirements for the maize based on the Malaysia Agriculture Research Development Institute (MARDI)’s recommendations were 60 kg ha^−1^ N, 60 kg ha^−1^ P_2_O_5_ and 40 kg ha^−1^ K_2_O. The chemical fertilizers used were urea (46% N), Egyptian Rock Phosphate (ERP) (28% P_2_O_5_) and muriate of potash (MOP) or KCl (60% K_2_O). For treatment 7 there was 100% fertilization based on MARDI’s recommended rates which when scaled down to per pot basis was equivalent to 1.44 g urea, 5.87 g ERP and 3.58 g MOP. These fertilizers were applied in two equal splits at 10 and 28 days after seeding (DAS). The combined effects of bacterial inoculums, compost and chemical fertilizer at 50% total fertilization requirement of maize was compared to no fertilization (Control), organic-chemical fertilization (OCF) at 50% total requirement and 100% total requirement using chemical fertilizer alone (Table 1).

Treatments 2 to 6 had the total fertilization level reduced to only 50% supplied mainly by a combination of compost and chemical fertilizer with/or without bacterial inoculants according to the treatment design. They were applied at 10 DAS only. The amounts of organic fertilizer (compost) and chemical fertilizers used in combination with treatments in this study were based on P content of the compost because it has been reported that basing organic fertilizer on N supply typically results in P addition in excess of crops needs [70]. Five grams of peat moss-based bacterial inoculums were inoculated into the pots according to the treatment design.

Similar amounts of the autoclaved carrier-based inocula were also added to all the treatments without bacterial inoculation so as to maintain similarity of soil texture and other properties for all the pots. There were seven replications for each treatment. The maize seeds were washed for a few rounds to remove any fungicide coatings, followed by surface disinfection using 0.1% sodium hypochlorite solution for 5 min before rinsing at least three times with distilled water to thoroughly remove sterilants. The seeds were then soaked in water for 24 hrs to aid seed germination and better establishment of seedlings upon seeding.

The soil in each pot was moistened up to 70% field capacity using tap water prior to sowing the germinated seeds. Four seeds were sown directly into planting holes at 4 cm soil depth per pot and then the holes were loosely covered with soil from the surface to enable rapid emergence of the seeds. At seven DAS the seedlings were thinned to two per pot so as to reduce competition among the plants. Soil moisture was maintained at field capacity using tap water. The plants were monitored up to tasselling stage at 56 DAS. Tasselling stage is the final development stage of maize before it reaches productive stage [71]. 

### 2.6. Biological and Chemical Analyses

Growth variables of maize were determined based on height using a measuring tape and stem diameter at 10 cm from soil surface using a digital vernier calliper at 56 DAS. Chlorophyll content was also determined using a Minolta’s SPAD-502 chlorophyll meter. The crops were harvested at 56 DAS. The maize plants were partitioned into leaves, stem and roots. The plant parts were then washed with tap water, rinsed using distilled water and blotted gently to remove contaminants such as soil residues and dust followed by oven-drying at 60 °C to constant weight. The dry weight of the plant parts was measured using a digital balance. The oven-dried plant samples were ground using a grinder before being analyzed for total N content using Kjeldahl method [64], and both P and K contents by dry ashing extraction [63], after which P was analyzed using the molybdenum blue method [67] and K by using an AAS. Nutrient uptake was calculated by multiplying nutrient concentration with the dry weight of the respective plant parts. Nutrient use efficiency was calculated according to the Pomares-Garcia and Pratt [72] equation:

Uptake = Nutrient concentration × dry weight (g)

% efficiency = (*A* − *B*)/*C* × 100

*A* = uptake with fertilizer; *B* = uptake without fertilizer; *C* = Total amount of fertilizer applied

Soil samples were also collected at 56 DAS for bacterial enumeration and the aforementioned soil analyses. Enumeration of cellulolytic, N-fixing and phosphate-solubilizing bacterial populations was also done at 30 DAS and it was determined from fresh soil samples using suspension dilution techniques on the aforementioned respective selective media under Section 2.1. 

### 2.7. Data Analysis

Treatment effects were ascertained using analysis of variance (ANOVA) and Tukey’s Test was used for comparison of treatment means. The Statistical Analysis System (SAS) version 9.2 was used for all statistical analysis at a significance level of *P* ≤ 0.05.

## 3. Results and Discussion

### 3.1. Interactions or Compatibility of Bacterial Isolates and Exponential Growth Equations of Bacterial Inoculants

Cross-streak assay between cellulolytic *Serratia nematodiphila* C46d, phosphate-solubilizing *Burkholderia cepacia* PB3 and N-fixing *Paraburkholderia nodosa* NB1 isolates in this study revealed that they were all compatible meaning that no antagonistic interactions existed between the isolates. This was indicated by the absence of an inhibition zone around the streaked colonies of these isolates. The compatibility or interaction test was done as a simple means to identify any antagonistic effect of the bacterial strains against each other prior to the application of the consortium inoculants in the pot trial.

The exponential growth curve of each of the selected bacterial inoculants was a linear regression [cfu mL^−1^ *vis a vis* optical density (OD) values]. The linear regression equations obtained for the isolates were as follows: 

*Serratia nematodiphila* C46d:

Log [cfu mL^−1^] = 0.84 *[OD] + 7.90, Ra^2^ = 0.95 **;

*Burkholderia cepacia* PB3:

Log [cfu mL^−1^] = 0.88 *[OD] + 7.87, Ra^2^ = 0.99 **;

*Paraburkholderia nodosa* NB1:

Log [cfu mL^−1^] = 1.76 *[OD] + 4.94, Ra^2^ = 0.91 **.

where Ra^2^ is adjusted R^2^, the coefficient of determination for each linear regression equation; ** indicates highly significant linear regression statistically. Both the high Ra^2^ values and highly significant regressions denote a high degree of data fit for the said equations.

Based on the equations obtained for the bacterial isolates, both *Serratia nematodiphila* C46d and *Burkholderia cepacia* PB3 had relatively the same exponential growth rates, whereas *Paraburkholderia nodosa* NB1 showed a slower log growth rate. The determination of the bacterial growth rate equation at the exponential phase was most important in this study to ensure their most active propagation or population growth and/or with lowest mortality rate for the purpose of harvesting as inoculums.

The mean content of the *Paraburkholderia nodosa* NB1, *Burkholderia cepacia* PB3 and *Serratia nematodiphila* C46d inoculants in the peat moss solid carrier were 3.13 × 10^8^ cfu mL^−1^ g^−1^, 2.99 × 10^8^ cfu mL^−1^ g^−1^ and 2.60 × 10^8^ cfu mL^−1^ g^−1^, respectively. These values were quantified based on treatments after the curing period and prior to inoculation for the pot trial.

### 3.2. Establishment and Population Dynamics of Cellulolytic, Nitrogen-Fixing and Phosphate-solubilizing Bacteria in the Maize Rhizosphere

**Figure 1:**
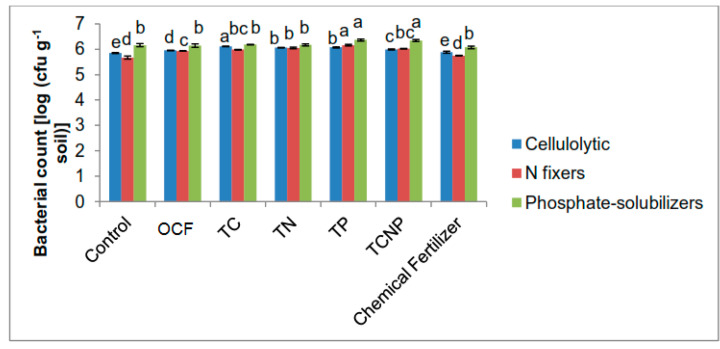
Population of cellulolytic, nitrogen-fixing and phosphate-solubilizing bacteria in the rhizosphere of maize at thirty days after sowing. Different letters for similar colored bars indicate significant difference between treatment means using Tukey’s test at *P* ≤ 0.05.

**Figure 2:**
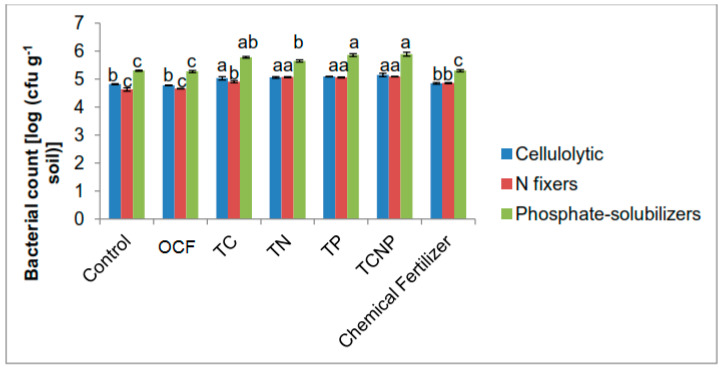
Population of cellulolytic, nitrogen-fixing and phosphate-solubilizing bacteria in the rhizosphere of maize at fifty-six days after sowing. Different letters for similar colored bars indicate significant difference between treatment means using Tukey’s test at *P* ≤ 0.05.

The study revealed generally higher functional bacterial populations for the inoculated treatments (Figure 1 and Figure 2) at both 30 and 56 DAS. At 30 DAS, the cellulolytic population was most abundant for the cellulolytic bacterial *S*. *nematodiphila* C46d inoculated treatment (TC) whereas phosphate-solubilizers (phosphate-solubilizing *B*. *cepacia* PB3 inoculated rhizospheres) were greater in number for treatments TP and TCNP (Figure 1). However, the N-fixing bacterial population was higher for treatment TP than for TN and TCNP treated with N-fixing *P*. *nodosa* NB1. This could be attributed to the slower growth of *P*. *nodosa* NB1, whereby its ability to compete with other indigenous rhizospheric microbes to colonize maize rhizospheres had not reached its optimum. Furthermore, during this early period of maize growth, the consortium bacterial treatment TCNP containing *S*. *nematodiphila* C46d, *P*. *nodosa* NB1 and *B. cepacia* PB3 may have not fully colonized the maize rhizospheres except for the *B*. *cepacia* PB3 population.

Significant population shifts in terms of higher abundance of functional bacterial populations were observed at 56 DAS. The pattern observed was that of cellulolytic bacterial types being higher in numbers at similar rates for all bacterial treatments whereas N-fixing populations showed dominant colonizations for treatments TN, TP and TCNP (Figure 2). Phosphate-solubilizers maintained dominant colonies for TP and TCNP. The colonization patterns of the introduced functional bacteria on maize as the host plant suggest that *B*. *cepacia* PB3 are aggressive colonizers, *P*. *nodosa* NB1 slow but effective colonizers and *S*. *nematodiphila* C46d effective colonizers for both single inoculation and consortium treatments but slower in colonization for the consortium treatment.

Plant growth-promoting rhizobacteria are known to comprise wide taxonomic diversity particularly within the Firmicutes and Proteobacteria phyla and are considered to be related to a wide range of host plant species [73,74] as indicated by the bacterial inoculant strains used in this study. In a related study the colonization of certain introduced microbes stimulated the propagation of other beneficial microbes and thereby exerted a synergistic effect on plant growth [48]. Root exudates released by plants can influence bacterial gene expression especially plant-beneficial properties encoded genes [75]. For example, in response to changes in root exudation, about one-third the level of C substances secreted from roots have been reported to have stimulated some *Azospirillum* PGPR [76]. This may be the reason for the higher populations of other functional bacteria for the different bacterial treatments. This was due to the co-predominance of the cellulolytic populations for the TN and TP treatments, respectively.

There were lower functional bacterial populations for the treatments without bacteria inoculants (Control, OCF and Chemical Fertilizer) than for the bacterial treatments. This suggests that the selected strains used for the bacterial treatments, especially *P. nodosa* NB1 and *B. cepacia* PB3, could affect or induce the propagation of their own kind and/or other functional bacterial populations in maize rhizospheres better than the indigenous populations which originally existed in the unsterilized soil used in the pot trial. 

### 3.3. Maize Plant Biomass, Height, Stem Diameter and Chlorophyll Content

The dry matter of the maize plants at 56 DAS ranged from 52.17 to 67.75 g plant^−1^ (Table 2). The uninoculated and unfertilized plants exhibited poor growth because of N, P and K nutrient deficiency. However, the non-sterilization of the soil could have resulted in the existence of an abundant native microflora which to some extent enhanced plant growth and nutrient uptake. In the course of this greenhouse pot study, moderate plant growth was observed for the rest of the treatments due to nutrients applied in chemical and/or organic forms.

With the combination of inoculation of functional bacteria, the fertilizer effect of both forms of the fertilizers at 50% plant requirement resulted in pronounced effects on growth variables such as total dry matter, height and chlorophyll content, similar to that with 100% recommended level of chemical fertilization (Table 2 and Table 3). Higher plant biomass with regards to combinations with bacterial treatments was achieved with TN and TP, whereby leaves and roots production for TN and stem and roots mass for TP were comparable to those of the Chemical Fertilizer treatments. For total maize biomass, the bacterial treatments did not show any significant effect with 50% fertilizer for OCF and the 100% Chemical Fertilizer treatment. The plant heights were also similar for all treatments although the plant heights for the TCNP and OCF treatments were not significantly different from the Control. The effects of the bacterial treatments on stem diameter were also not significantly different although lower than that for the Chemical Fertilizer treatments but higher than that for OCF. TN was the only bacterial treatment which showed similar chlorophyll content with that of the Chemical Fertilizer treatment whereas the rest of other bacterial treatments showed effects similar to that for OCF. 

The unexpected higher biomass and height of the maize plants for the Control and OCF treatments without bacterial inoculation could be attributed to the presence of native microbes in the unsterilized soil and this might have contributed to nutrient bioavailability and uptake in the rhizosphere. Plant growth inducement due to microbe inoculation in soil can result in improved nutrient acquisition due to plant root volume expansion and also increase the plants’ photosynthetic functions. However, there are cases where plant growth may be reduced resulting in a decline in nutrient uptake [77].

### 3.4. Nitrogen, Phosphorus and Potassium Concentrations in Maize Plant Parts

With the exception of the concentration of N in the stem for the TC treatment, the effects of the bacterial treatments on N and K concentrations in leaves and stem respectively, were similar (Table 4). Except for the K concentration in the roots for the TP treatment which was different from those for TC and TCNP, the N, P and K concentrations for the other treatments were similar. Phosphorus concentrations in the stem were similar for all treatments except for TC where the effect was different from that for TCNP. The bacterial and OCF treatments did not show significant effects on N concentrations regardless of the maize plant parts. Further, N concentration in roots for these treatments were comparable to that of the Chemical Fertilizer treatment but N concentrations in the leaves and stems for the bacterial and OCF treatments were lower than that for the Chemical Fertilizer treatment. TN showed better effects on leaf P concentration compared to the other treatments including the Chemical Fertilizer treatment.

For K concentrations in leaves and stems, the bacterial treatments showed similar results as that for the OCF and Control treatments but had lower concentrations than that for the Chemical Fertilizer treatment. Although the plant dry matter, height and stem diameter for the Control and OCF treatments were generally lower than that of the bacterial treatments, the content of some nutrient in some plant parts of the control plants were higher compared with some of the bacterial treatments. This is because the control plants may have attained critical nutrient concentration levels. At critical concentrations, growth does not increase to balance the excess nutrient uptake [78]. 

### 3.5. Nitrogen, Phosphorus and Potassium Uptake in Maize Plant Parts

**Figure 3:**
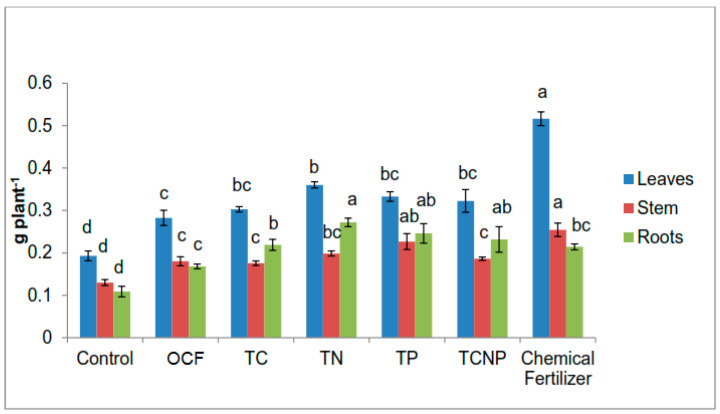
Effect of treatments on nitrogen uptake of maize plants. Different letters for similar colored bars indicate significant difference between treatment means using Tukey’s test at *P* ≤ 0.05. Nitrogen uptake was calculated by multiplying nitrogen concentration with the dry weight of the respective plant parts.

All bacterial treatments showed a relatively similar pattern in terms of N uptake for roots but the uptake values for the roots were higher than for the stems (Figure 3). Such a pattern is in contrast with that exhibited by the Control, OCF and Chemical Fertilizer treatments. These results suggest that the colonization of the inoculated bacterial strains used in this study stimulated growth of the maize plants’ roots via increased N acquisition, especially for the TN treated plants which showed a 27% higher roots N uptake than that for the Chemical Fertilizer treatment (Figure 3). However, all of the treatments showed a common trend whereby N uptake in leaves was higher than that in the stems and roots. In terms of stem N uptake, there was no significant difference between the OCF and bacterial treatments except for TP, which showed similar effects as that of the Chemical Fertilizer treatment, but significantly higher than that of OCF (Figure 3). 

**Figure 4:**
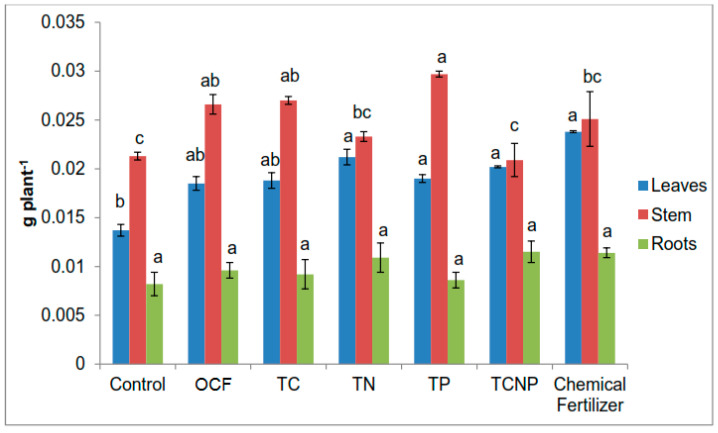
Effect of treatments on phosphorus uptake in maize plants. Different letters for similar colored bars indicate significant difference between treatment means using Tukey’s test at *P* ≤ 0.05. Phosphorus uptake was calculated by multiplying phosphorus concentration and the dry weight of the respective plant parts.

Except for TP whose effect on P uptake in stems was superior to that of the Chemical Fertilizer treatment by 18%, the rest of the treatments showed similar effects on P uptake in the stems (Figure 4). Regardless of treatment, there was no significant difference in P uptake in the roots of the maize plants. Similarly, P uptake in the leaves also demonstrated generally comparable outcome with the exception that treatments with TN, TP, TCNP and Chemical Fertilizer were significantly higher than Control (Figure 4).

**Figure 5:**
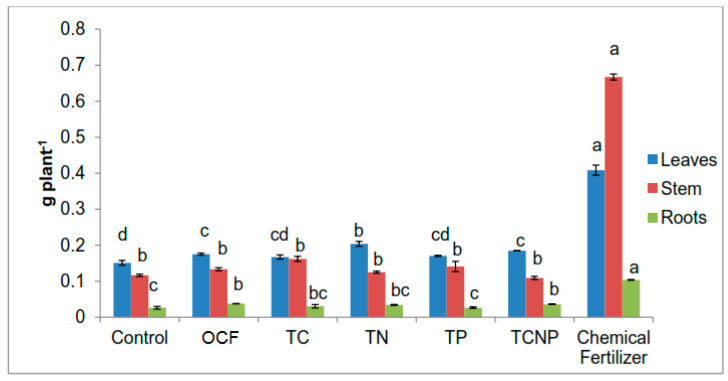
Effect of treatments on potassium uptake in maize plants. Different letters for similar colored bars indicate significant difference between treatment means using Tukey’s test at *P* ≤ 0.05. Potassium uptake was calculated by multiplying potassium concentration with the dry weight of the respective plant parts.

For plant K uptake, there were noticeable differences in effects between the bacterial treatments and the Chemical Fertilizer treatment (Figure 5). However, among the bacterial treatments, TN treated plants showed a significant improvement in K uptake in leaves (116.4 % higher) than that for OCF, followed by the Chemical Fertilizer treatment. However no significant effect on K uptake was observed in roots irrespective of the treatment (Figure 5).

Generally, treatments with TN showed consistent significant effects on N, P and K uptake in the leaves and roots. Mantelin and Touraine [79] stated that PGPR exerts an influence on plant nutrition based on the impact following plant nutrient uptake and/or plant growth rate. It is generally believed that improved nutrient uptake is caused by enhanced root surface area as stimulated by PGPR activities [75]. For instance, many inocula reported in other studies as N fixers, phosphate-solubilizers and plant growth stimulators have attributed beneficial effects to the media through the secretion of substances such as phospholipids, vitamins, phytohormones, siderophores and antibiotics among others [80,81,82,83].

### 3.6. Nitrogen, Phosphorus and Potassium Use Efficiency in Maize Plant Parts

Effects of TN on N and P use efficiencies in leaves and roots and their respective totals are presented in Table 5 and Table 6. Nitrogen use efficiency in roots and total maize parts for TN were better than that for the Chemical Fertilizer treatments although their effects on leaves and stems were similar (Table 5).

For P use efficiency, TN treated plants showed better results for the leaves, roots and total efficiency than that for the Chemical Fertilizer treated plants, but these treatments had similar effects on the maize stems (Table 6). The other bacterial treatments (TC and TCNP, as well as TN) improved total N use efficiency and leaf P use efficiency as compared to the OCF treatment. The TP treatment also improved N use efficiencies in stem, roots and total efficiency over that of the Chemical Fertilizer treatment, as well as P use efficiencies in stem and leaf over the OCF treatment.

Potassium use efficiency in the bacterial treatments was lower than that for the Chemical Fertilizer treatment (Table 7). Nonetheless, among the bacterial treatments, TN showed the highest K use efficiency and it was the only bacterial treatment which showed a higher K use efficiency than the OCF treatment. The rest of the other bacterial treatments generally showed lower results for K use efficiency compared with the OCF treatment.

These results suggest that the bacterial treatments combination with fertilization could compensate at least half the N and P fertilizer amounts as compared to the conventional approach of using chemical fertilizers alone.

According to Sheng and Huang [84], K content reduces easily in soils, due to many factors which include crop uptake, leaching, runoff and soil erosion. In this study, the application of K fertilizer mainly in the form of soluble K, in combination with non-readily available and slow release source of K from compost, especially for OCF and all the bacterial treatments at half the requirement level might have been leached during watering. This explains the lower K uptake by the affected plants.

For both nutrient uptake and use efficiency, treatment TC and especially the consortium bacterial treatment TCNP showed poorer results compared with the single bacterial treatments, namely TN and TP. These different outcomes of nutrient assimilation from using different strains for co-inoculation can have effects on plant development. For example, Sarr et al. [85] reported that co-inoculation of *Ralstonia* sp. TSC1 with bradyrhizobial strains TSC10 and DTB4 produced markedly increased nodule dry weight, whereas application of *Ralstonia* sp. TSC1 with bradyrhizobium strain DTC9 led to significantly reduced nodulation and N_2_ fixation, suggesting that *Ralstonia* sp. TSC1 may be antagonistic to strain DTC9. Recent studies indicate that particular bacterial isolates flourish better when mixed but there are many situations where the bacteria impose detrimental effects on each other [86] which leads to poor plant development.

In the presence of on antagonistic microflora, rhizobacteria populate all ecological niches of plant roots at all plant developmental stages [87]. Competition amongst inoculated strains is common as it generally occurs amongst soil-borne microbes [88]. Hence, this fairly neutral outcome limits the application of consortia isolates *Paraburkholderia nodosa* NB1, *Burkholderia cepacia* PB3 and *Serratia nematodiphila* C46d simultaneously to improve maize productivity.

According to Vacheron et al. [75], interactions amongst the PGPR strains co-habiting within a rhizosphere milieu could occur within a functional group and may also prevail between distinct PGPR functional groups. In the latter relationships, they could involve competitive and suppressive effects [89], positive signaling [90], signal jamming [91] and more indirect mechanisms such as alterations of root exudates [92,93]. Such relationships may likely affect regulation of spatial colonization distribution of PGPR on roots [89], which influences PGPR performance [94]. This results in synergistic or antagonistic effects from members of different PGPR functional groups, particularly in consortia treatments. Therefore, the expression of plant-beneficial functions, particularly biocontrol properties in PGPR, could be compromised by other members of the bacterial rhizosphere community [75].

Phytostimulants involving bacterial species from the genera *Burkholderia* besides *Azospirillum, Arthrobacter* and *Achromobacter* as suggested by several studies [95], have resulted in shoot and root weight increment and higher nutrient uptake of maize plants. Their beneficial effects might be due to other activities such as phosphate-solubilization as well as other non-evaluated PGPR attributes such as phytohormone production, like IAA that promotes plant growth.

### 3.7. Soil Properties

**Figure A1:**
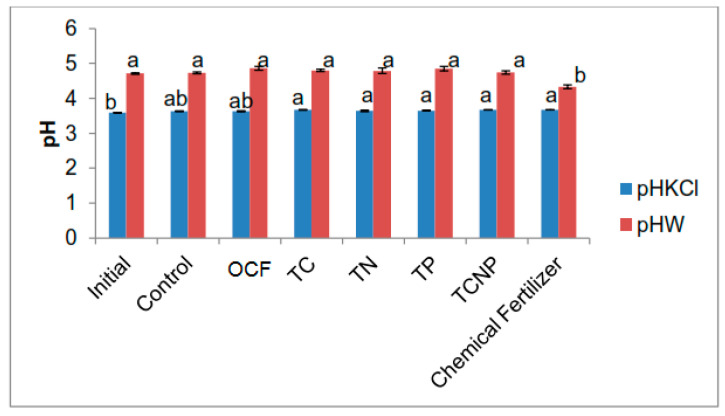
Effect of treatments on soil pH in KCl and water at fifty-six days after sowing. Different letters for similar colored bars indicate significant difference between treatment means using Tukey’s test at *P* ≤ 0.05.

Soil treated with chemical fertilizer alone had a lower pH in water (more acidic) compared with those of the other treatments but soil pH generally increased after bacterial treatments and chemical fertilization (Figure A1). Soil pH is an important soil property which can be used to indicate the availability of plant nutrients as most crops grow optimally at neutral soil pH [96]. Soil pH in water measures acidity in the soil solution whereas pH in KCl also measures the reserve acidity in the soil colloids [96]. The KCl method can be used to overcome the presence of soluble salt content in the soil (especially from the use of chemical fertilizers which can generally reduce the soil pH) instead of distilled water [97]. Plant macronutrients include elements such as N, P, K, Ca and Mg which can be derived both from the soil solution and soil organic matter [96]. 

Soil exchangeable K and available P were higher for the chemical fertilizer treatment alone (Table A2) but total N, ammonium and nitrate contents were similar regardless of the treatment (Table A3). The method of application and form of nutrient fertilizers especially macronutrients have a significant impact on soil pH. Nitrogen fertilizers have a major influence on soil pH changes because root uptake of nitrate produced from ammonium of N fertilizers results in the release of one H^+^ ion. This process is one of the causes of soil acidification in the plant roots rhizosphere, especially where there is poor uptake of K, Mg and Ca for corresponding release of OH^-^ ions to neutralize the H^+^ ions [98]. Thus, the lower soil pH due to chemical fertilization alone could be partly related to lower nutrient uptake and use efficiency, especially of N.

Bacterial treatments TN and TP showed higher soil exchangeable Ca compared to the other treatments (Table A2). Bacteria might have also contributed to the increased pH in the soils with TN and TP as indicated by the higher soil exchangeable Ca in this study. Despite no significant changes in pH, the particularly high exchangeable Ca for TN could be attributed to the stimulating effects of *Paraburkholderia nodosa* NB1 on the decomposition activities of the other microbes, thus causing release of Ca from soil organic matter and especially from the added compost. Furthermore, it has been reported that a group of bacteria may produce amino acids and an array of growth factors that attract other bacterial groups which improves nutrient utilization [99]. *Paraburkholderia nodosa* NB1 strain possesses phosphate-solubilizing ability to liberate Ca from the ERP (phosphate fertilizer) used in this study. 

Soil CEC generally improved for all treatments when compared to the control and pre-planting soils (Table A2). Soil CEC reflects the total capacity of a soil to hold exchangeable cations which affects the ability of the soil to retain essential nutrients and buffering against soil acidification [96]. No significant differences resulted for soil total N, ammonium and nitrate between the various treatments (Table A3). For soil total C and organic matter contents, the TN treatment effect was significantly higher than that of the Chemical Fertilizer treatment (Table A4). Enhanced microbiological activities and improved soil physical and chemical properties are achieved with the addition of organic matter although the organic matter has relatively low N. Biological N fixation remains one of the most essential means to contribute N to soils, particularly along with organic matter [100]. Bar-Tal et al. [101] also reported the beneficial influence of compost on crops only upon application of extra N-fertilizer. This is evident or consistent with the highest total N use efficiency for the TN plants (refer to Table 5).

### 3.8. Interactions between Bacterial, Plant and Soil Parameters

Maize plant biomass showed strong relationships with N and P uptake despite no significant relationship was observed between soil organic matter and plant biomass (Table 8). The functional bacterial populations consisting of cellulolytic, N fixers and phosphate-solubilizers were also strongly correlated with one another which explains their synergistic effects. This finding corroborates that of Kuan et al. [102] who also found positive correlation among rhizospheric bacterial populations. Only the N-fixing populations seemed to show an influence on plant biomass, as observed in their strong relationship with plant N uptake and the positive relationship with P uptake.

## 4. Conclusions

Beneficial bacterial applications in 50% combined fertilization showed both comparable and higher maize nutrient use efficiencies especially N and P utilization than that with full chemical fertilization. Nitrogen-fixing rhizobacteria had significant effects on maize biomass, plant N and P uptake. Treatments with *Paraburkholderia nodosa* NB1 improved soil total C and organic matter contents. Moreover, it was the only bacterial treatment that showed higher K use efficiency over non-inoculated combined-fertilizer treatment. *Paraburkholderia nodosa* NB1 and *Burkholderia cepacia* PB3 showed positive effects on maize biomass but their effects were more pronounced in improving N and P use efficiencies over the full chemical fertilization treatment. This indicates that *Paraburkholderia nodosa* NB1 and *B. cepacia* PB3 are capable of stimulating other beneficial functional bacterial growth in the soil rhizosphere to enhance maize growth. The combined strains or consortia had different influences on the functional rhizobacterial populations and maize plants’ development. *Serratia nematodiphila* C46d and consortium strains as inocula in this study showed no significant effects on the nutrient assimilation and growth of maize, which is attributed to competition in the maize rhizosphere. Using PGPR isolates with compost and chemical fertilizer proved sustainable for INM with minimal use of chemical fertilizers. *Paraburkholderia nodosa* NB1 and *B. cepacia* PB3 have good potential in future field trials for them to be referred to as bio-fertilizers. However, it should be noted that the *Burkholderia cepacia* PB3 belongs to the Bcc group of strains which may be pathogenic to humans. Therefore, stringent assessment of potential risks may be required for future development of agricultural and/or biotechnological applications using this strain.

## Figures and Tables

**Table 1:** Information on the treatments used in the greenhouse study.

Treatment	1	2	3	4	5	6	7
With bacterial inoculation	No	No	Yes	Yes	Yes	Yes	No
Bacterial species added	-	-	Cellulolytic SN ^a^	N-fixing PN ^b^	Phosphate- solubilizing BC ^c^	SN+PN+ BC	-
Fertilizer used	-	OF ^d^ + CF ^e^	OF + CF	OF + CF	OF + CF	OF + CF	CF
Total fertilization level	0%	50%	50%	50%	50%	50%	100%
**Code**	**Control**	**OCF**	**TC**	**TN**	**TP**	**TCNP**	**Chemical Fertilizer**

^a^ SN—*Serratia nematodiphila* C46d, ^b^ PN—*Paraburkholderia nodosa* NB1, ^c^ BC—*Burkholderia cepacia* PB3, ^d^ OF—organic fertilizer (compost), ^e^ CF—chemical fertilizer.

**Table 2:** Dry weight of leaves, stem and roots of maize plants at fifty-six days after sowing.

Treatment	Mean Value ± S.E. of Dry Weight (g Plant^−1^)			
	Leaves	Stem	Roots	Total Biomass
Control	19.42 ^e^ ± 0.38	20.31 ^c^ ± 1.16	0.63 ^c^ ± 0.36	52.17 ^c^ ± 2.01
OCF	21.66 ^d^ ± 0.52	22.97 ^bc^ ± 0.60	0.84 ^b^ ± 0.28	61.66 ^b^ ± 0.62
TC	22.65 ^cd^ ± 0.37	27.97 ^a^ ± 1.00	0.65 ^c^ ± 0.38	66.78 ^ab^ ± 1.09
TN	24.74 ^ab^ ± 1.12	23.90 ^abc^ ± 1.03	0.84 ^b^ ± 0.24	67.07 ^a^ ± 0.19
TP	23.18 ^bcd^ ±0.06	26.70 ^ab^ ± 2.47	0.82 ^b^ ± 0.70	67.75 ^a^ ± 3.25
TCNP	24.10 ^bc^ ± 0.55	23.05 ^bc^ ± 0.31	0.82 ^b^ ± 0.73	64.39 ^ab^ ± 2.71
Chemical Fertilizer	26.52 ^a^ ± 0.97	25.53 ^ab^ ± 1.08	1.00 ^a^ ± 0.79	66.79 ^ab^ ± 0.85

Different letters within each column indicate significant difference between means using Tukey’s test at *P* ≤ 0.05. S.E. is standard error.

**Table 3:** Height, stem diameter and chlorophyll content of maize plants at fifty-six days after sowing.

Treatment	Height (cm)	Stem Diameter (mm)	Chlorophyll Content (SPAD value)
	Mean Value ± S.E.		
Control	102.10 ^b^ ± 2.13	11.90 ^e^ ± 0.04	29.43 ^b^ ± 0.20
OCF	110.34 ^ab^ ± 2.07	12.54 ^d^ ± 0.20	33.43 ^a^ ± 0.42
TC	118.58 ^a^ ± 0.23	12.88 ^d^ ± 0.07	33.38 ^a^ ± 0.17
TN	120.14 ^a^ ± 3.75	13.31 ^c^ ± 0.11	33.96 ^a^ ± 0.26
TP	117.47 ^a^ ± 3.59	13.39 ^c^ ± 0.05	34.02 ^a^ ± 0.31
TCNP	113.28 ^ab^ ± 0.79	13.99 ^b^ ± 0.19	33.53 ^a^ ± 0.22
Chemical Fertilizer	118.18 ^a^ ± 4.44	14.64 ^a^ ± 0.09	34.28 ^a^ ± 0.13

Different letters within each column indicate significant difference between means using Tukey’s test at *P* ≤ 0.05. S.E. is standard error.

**Table 4:** Effect of treatments on the concentrations of nitrogen, phosphorus and potassium in leaves, stem and roots of maize plant at fifty-six days after sowing.

**N (%)** **Mean Value ± S.E.**			
**Treatment**	**Leaves**	**Stem**	**Roots**
Control	0.98 ^c^ ± 0.04	0.63 ^c^ ± 0.08	0.93 ^b^ ± 0.06
OCF	1.47 ^b^ ± 0.00	0.84 ^b^ ± 0.00	1.21 ^a^ ± 0.12
TC	1.31 ^b^ ± 0.05	0.65 ^c^ ± 0.02	1.31 ^a^ ± 0.06
TN	1.49 ^b^ ± 0.05	0.84 ^b^ ± 0.07	1.26 ^a^ ± 0.04
TP	1.49 ^b^ ± 0.05	0.82 ^b^ ± 0.02	1.28 ^a^ ± 0.08
TCNP	1.47 ^b^ ± 0.11	0.82 ^b^ ± 0.05	1.35 ^a^ ± 0.02
Chemical Fertilizer	1.84 ^a^ ± 0.14	1.00 ^a^ ± 0.02	1.31 ^a^ ± 0.02
**P (%)** **Mean Value ± S.E.**			
**Treatment**	**Leaves**	**Stem**	**Roots**
Control	0.073 ^c^ ± 0.005	0.107 ^ab^ ± 0.006	0.093 ^a^ ± 0.004
OCF	0.082 ^bc^ ± 0.001	0.104 ^abc^ ± 0.002	0.086 ^ab^ ± 0.004
TC	0.088 ^ab^ ± 0.004	0.095 ^d^ ± 0.002	0.056 ^c^ ± 0.009
TN	0.094 ^a^ ± 0.002	0.099 ^bcd^ ± 0.002	0.067 ^abc^ ± 0.005
TP	0.082 ^bc^ ± 0.000	0.097 ^cd^ ± 0.001	0.048 ^c^ ± 0.003
TCNP	0.082 ^bc^ ± 0.002	0.106 ^abc^ ± 0.010	0.062 ^bc^ ± 0.013
Chemical Fertilizer	0.076 ^bc^ ± 0.003	0.111 ^a^ ± 0.004	0.069 ^abc^ ± 0.002
**K (%)** **Mean Value ± S.E.**			
**Treatment**	**Leaves**	**Stem**	**Roots**
Control	0.72 ^b^ ± 0.05	0.57 ^b^ ± 0.03	0.27 ^b^ ± 0.01
OCF	0.85 ^b^ ± 0.02	0.56 ^b^ ± 0.02	0.26 ^bc^ ± 0.00
TC	0.76 ^b^ ± 0.00	0.48 ^b^ ± 0.02	0.21 ^cd^ ± 0.05
TN	0.84 ^b^ ± 0.02	0.52 ^b^ ± 0.02	0.18 ^de^ ± 0.03
TP	0.78 ^b^ ± 0.04	0.54 ^b^ ± 0.01	0.14 ^e^ ± 0.01
TCNP	0.78 ^b^ ± 0.02	0.50 ^b^ ± 0.03	0.20 ^d^ ± 0.01
Chemical Fertilizer	1.61 ^a^ ± 0.11	2.79 ^a^ ± 0.09	0.64 ^a^ ± 0.01

Different letters within each column indicate significant difference between means using Tukey’s test at *P* ≤ 0.05. S.E. is standard error of the mean.

**Table 5:** Effect of treatments on nitrogen use efficiency in maize plant parts.

Treatment	N Use Efficiency (%) Mean Value ± S.E.			
	Stem	Leaves	Roots	Total
OCF	16.61 ^cd^ ± 0.59	26.99 ^c^ ± 5.14	21.41 ^cd^ ± 1.69	59.96 ^e^ ± 1.23
TC	13.72 ^d^ ± 1.40	33.07 ^bc^ ± 1.91	33.17 ^bc^ ± 3.91	80.77 ^d^ ± 3.62
TN	21.99 ^b^ ± 1.81	54.24 ^a^ ± 2.20	52.60 ^a^ ± 3.00	128.83 ^a^ ± 2.14
TP	34.71 ^a^ ± 0.81	42.16 ^ab^ ± 2.60	36.19 ^b^ ± 7.06	99.86 ^b^ ± 3.76
TCNP	16.91 ^cd^ ± 1.17	39.01 ^bc^ ± 7.11	30.07 ^bc^ ± 3.87	89.90 ^c^ ± 3.45
Chemical Fertilizer	20.98 ^bc^ ± 1.37	44.66 ^ab^ ± 2.44	15.87 ^d^ ± 1.25	79.28 ^d^ ± 0.51

Different letters within each column indicate significant difference between means using Tukey’s test at *P* ≤ 0.05. S.E. is standard error.

**Table 6:** Effect of treatments on phosphorus use efficiency in maize plant parts.

Treatment	P Use Efficiency (%) Mean Value ± S.E.			
	Stem	Leaves	Roots	Total
OCF	0.92 ^bc^ ± 0.35	0.86 ^c^ ± 0.29	0.40 ^b^ ± 0.21	2.72 ^abc^ ± 0.12
TC	1.74 ^ab^ ± 0.11	1.40 ^ab^ ± 0.21	−0.14 ^c^ ± 0.03	2.49 ^bc^ ± 0.08
TN	0.56 ^c^ ± 0.12	1.88 ^a^ ± 0.06	1.35 ^a^ ± 0.22	3.34 ^a^ ± 0.26
TP	2.30 ^a^ ± 0.12	1.46 ^a^ ± 0.12	0.44 ^b^ ± 0.17	3.01 ^ab^ ± 0.53
TCNP	0.63 ^c^ ± 0.28	1.48 ^a^ ± 0.03	0.63 ^b^ ± 0.11	2.40 ^bc^ ± 0.04
Chemical Fertilizer	0.52 ^c^ ± 0.38	0.93 ^bc^ ± 0.01	0.44 ^b^ ± 0.07	2.00 ^c^ ± 0.21

Different letters within each column indicate significant difference between means using Tukey’s test at *P* ≤ 0.05. S.E. is standard error.

**Table 7:** Effect of treatments on potassium use efficiency in maize plant parts.

Treatment	K Use Efficiency (%)Mean Value ± S.E.			
	Stem	Leaves	Roots	Total
OCF	0.91 ^b^ ± 0.28	1.65 ^c^ ± 0.18	0.76 ^b^ ± 0.05	3.32 ^c^ ± 0.10
TC	1.11 ^b^ ± 0.48	1.10 ^c^ ± 0.38	0.43 ^bc^ ± 0.33	2.88 ^d^ ± 0.23
TN	0.61 ^bc^ ± 0.20	3.57 ^b^ ± 0.40	0.53 ^b^ ± 0.09	4.29 ^b^ ± 0.09
TP	0.49 ^bc^ ± 0.30	1.32 ^c^ ± 0.10	0.02 ^c^ ± 0.14	1.82 ^e^ ± 0.23
TCNP	−0.47 ^c^ ± 0.27	2.39 ^bc^ ± 0.01	0.76 ^b^ ± 0.05	2.03 ^e^ ± 0.11
Chemical Fertilizer	34.80 ^a^ ± 0.47	14.46 ^a^ ± 0.69	4.35 ^a^ ± 0.14	53.08 ^a^ ± 0.14

Different letters within each column indicate significant difference between means using Tukey’s test at *P* ≤ 0.05. S.E. is standard error.

**Table 8:** Pearson’s correlation matrix for maize plant, soil and bacterial parameters.

	Biomass	N Uptake	P Uptake	K Uptake	Organic Matter	Avail.P	Soil Total N	Avail. K	Cellulo-Lytic Pop.	N Fixers	P-Sols
Biomass	1.00										
N Uptake	0.81 **	1.00									
P Uptake	0.80 **	0.82 **	1.00								
K Uptake	NS	0.63 **	NS	1.00							
Organic matter	NS	NS	NS	NS	1.00						
Available P	NS	0.62 **	NS	0.94 **	NS	1.00					
Soil total N	NS	NS	NS	NS	NS	NS	1.00				
Available K	NS	0.57 *	NS	0.97 **	NS	0.91 **	NS	1.00			
Cellulolytic Pop.	NS	NS	NS	NS	NS	NS	NS	NS	1.00		
N fixers	0.57 *	0.66 **	0.57 *	NS	NS	NS	NS	NS	0.89 **	1.00	
Phosphate-solubilizers	NS	NS	NS	NS	NS	NS	NS	NS	0.90 **	0.81 **	1.00

Levels of significance: * *P ≤* 0.05, ** *P ≤* 0.01. NS denotes non-significant relationship.

## Appendix A

**Table A1:** Selected chemical properties of Bekenu series.

Property	Value Obtained	Reported Data Range *
	(Mean ± S.E.)	
pH_Water_	4.71 (± 0.02)	4.60–4.90
pH_KCl_	3.59 (± 0.01)	3.80–4.00
Exchangeable potassium (cmol_c_ kg^−1^)	0.10 (± 0.00)	0.05–0.19
Exchangeable calcium (cmol_c_ kg^−1^)	6.68 (± 0.10)	0.01
Exchangeable magnesium (cmol_c_ kg^−1^)	45.33 (± 0.30)	0.07–0.21
CEC (cmol_c_ kg^−1^)	8.13 (± 0.53)	3.86–8.46
Total nitrogen (%)	0.13 (± 0.02)	0.04–0.17
Total carbon (%)	2.60 (± 0.04)	0.57–2.51
Organic matter (%)	4.48 (± 0.07)	nd
Carbon/nitrogen ratio	20.45 (± 2.78)	3.35–62.75
Available phosphorus (mg kg^−1^)	6.22 (± 0.16)	nd
Exchangeable ammonium (mg kg^−1^)	42.73 (± 0.70)	nd
Available nitrate (mg kg^−1^)	1.40 ^(^± 0.00)	nd

S.E. is standard error. * Reported data range [69]. CEC is cation exchange capacity. nd is not determined.

**Table A2:** Effect of treatments on soil exchangeable potassium, exchangeable calcium, cation exchange capacity and available phosphorus contents.

Treatment	Exchangeable K	Exchangeable Ca	CEC	Available P (mg kg^−1^)
	----------------------------[cmol (+) kg^−1^]------------------------------------			
	Mean Value ± S.E.			
Initial	0.100 ^b^ ± 0.001	0.033 ^d^ ± 0.0003	8.13 ^d^ ± 0.53	6.22 ^b^ ± 0.16
Control	0.041 ^c^ ± 0.002	0.058 ^c^ ± 0.006	8.45 ^cd^ ± 0.10	3.76 ^b^ ± 0.10
OCF	0.041 ^c^ ± 0.002	0.056 ^c^ ± 0.006	12.00 ^a^ ± 1.25	3.72 ^b^ ± 0.27
TC	0.044 ^c^ ± 0.002	0.067 ^c^ ± 0.001	10.98 ^ab^ ± 0.63	4.75 ^b^ ± 0.21
TN	0.044 ^c^ ± 0.002	0.090 ^a^ ± 0.010	10.53 ^ab^ ± 0.43	5.21 ^b^ ± 1.26
TP	0.043 ^c^ ± 0.003	0.078 ^ab^ ± 0.005	10.08 ^bc^ ± 0.41	4.82 ^b^ ± 0.87
TCNP	0.044 ^c^ ± 0.001	0.057 ^c^ ± 0.003	9.92 ^bc^ ± 0.20	3.52 ^b^ ± 0.21
Chemical Fertilizer	0.204 ^a^ ± 0.025	0.071 ^bc^ ± 0.003	10.18 ^b^ ± 0.32	14.21 ^a^ ± 1.59

Different letters within each column indicate significant difference between means using Tukey’s test at *P* ≤ 0.05. S.E. is standard error.

**Table A3:** Effect of treatments on soil total nitrogen, ammonium and nitrate contents.

Treatment	Total N (%)	Ammonium (mg kg^−1^)	Nitrate (mg kg^−1^)
	Mean Value ± S.E.		
Control	0.093 ^a^ ± 0.012	27.32 ^b^ ± 2.10	1.40 ^a^ ± 0.00
OCF	0.097 ^a^ ± 0.013	43.90 ^a^ ± 1.24	1.40 ^a^ ± 0.00
TC	0.107 ^a^ ± 0.003	42.03 ^a^ ± 2.14	1.40 ^a^ ± 0.00
TN	0.107 ^a^ ± 0.003	42.96 ^a^ ± 2.03	1.40 ^a^ ± 0.00
TP	0.100 ^a^ ± 0.010	41.56 ^a^ ± 1.87	1.40 ^a^ ± 0.00
TCNP	0.117 ^a^ ± 0.007	44.83 ^a^ ± 1.40	1.40 ^a^ ± 0.00
Chemical Fertilizer	0.093 ^a^ ± 0.012	43.43 ^a^ ± 2.14	1.40 ^a^ ± 0.00

Different letters within each column indicate significant difference between means using Tukey’s test at *P* ≤ 0.05. S.E. is standard error.

**Table A4:** Effect of treatments on soil total carbon and organic matter contents.

Treatment	Total C (%)	Organic Matter (%)	
	Mean Value ± S.E.		
Control	2.79 ^bc^ ± 0.02	4.81 ^bc^ ± 0.03	
OCF	2.91 ^ab^ ± 0.14	5.01 ^ab^ ± 0.24	
TC	2.83 ^ab^ ± 0.04	4.87 ^ab^ ± 0.07	
TN	3.00 ^a^ ± 0.01	5.16 ^a^ ± 0.01	
TP	2.77 ^bc^ ± 0.04	4.78 ^bc^ ± 0.07	
TCNP	2.78 ^bc^ ± 0.04	4.79 ^bc^ ± 0.06	
Chemical Fertilizer	2.74 ^bc^ ± 0.02	4.71 ^bc^ ± 0.03	

Different letters within each column indicate significant difference between means using Tukey’s test at *P* ≤ 0.05. S.E. is standard error.
